# Transplantation of Donor‐Origin Mouse Embryonic Stem Cell‐Derived Thymic Epithelial Progenitors Prevents the Development of Chronic Graft‐versus‐Host Disease in Mice

**DOI:** 10.5966/sctm.2016-0012

**Published:** 2016-08-02

**Authors:** Rong Hu, Yalan Liu, Min Su, Yinhong Song, Debra Rood, Laijun Lai

**Affiliations:** ^1^ Department of Allied Health Sciences, University of Connecticut, Storrs, Connecticut, USA; ^2^ Guizhou Medical University, Guizhou, People's Republic of China; ^3^ University of Connecticut Stem Cell Institute, University of Connecticut, Storrs, Connecticut, USA

**Keywords:** Chronic graft‐versus‐host disease, Thymic epithelial progenitors, Thymic epithelial cells, Embryonic stem cells, Tolerance induction

## Abstract

Allogeneic hematopoietic stem cell transplantation (HSCT) is a potentially curative therapy for many malignant and nonmalignant diseases. However, chronic graft‐versus‐host disease (cGVHD) remains a significant cause of late morbidity and mortality after allogeneic HSCT. cGVHD often manifests as autoimmune syndrome. Thymic epithelial cells (TECs) play a critical role in supporting negative selection and regulatory T‐cell (Treg) generation. Studies have shown that damage in TECs is sufficient to induce cGVHD. We have previously reported that mouse embryonic stem cells (mESCs) can be selectively induced to generate thymic epithelial progenitors (TEPs) in vitro. When transplanted in vivo, mESC‐TEPs further develop into TECs that support T‐cell development. We show here that transplantation of donor‐origin mESC‐TEPs into cGVHD recipients induces immune tolerance to both donor and host antigens and prevents the development of cGVHD. This is associated with more TECs and Tregs. Our results suggest that embryonic stem cell‐derived TEPs may offer a new tool to control cGVHD. Stem Cells Translational Medicine *2017;6:121–130*

**Significance Statement**

Allogeneic hematopoietic stem cell transplantation (HSCT) is widely used in the treatment of many malignant and nonmalignant diseases. However, chronic graft‐versus‐host disease (cGVHD) remains a significant cause of late morbidity and mortality following allogeneic HSCT. This study shows that transplantation of mouse embryonic stem cell (ESC)‐derived thymic epithelial progenitors (TEPs) into cGVHD recipient mice prevents the development of cGVHD. Results suggest that generation of TEPs from human ESCs have the potential to provide a novel approach to prevent the development of cGVHD in patients after allogeneic HSCT.

## Introduction

Allogeneic hematopoietic stem cell transplantation (HSCT) is widely used in the treatment of a variety of diseases, including hematopoietic diseases, congenital immunodeficiency, autoimmune diseases, and solid tumors. However, chronic graft‐versus‐host disease (cGVHD) remains as a common and serious complication following allogeneic HSCT 1 2 3. Based on different clinical manifestations and histopathology, GVHD can be divided into acute GVHD and cGVHD. The latter typically manifests as an autoimmune syndrome. Current therapies to prevent or treat cGVHD often rely on generalized immunosuppression. Despite achieving some success, immunosuppressive strategies often lead to the development of immune deficiency with a consequence of a high rate of opportunistic infections and the occurrence or relapse of cancers 1, 2.

Many studies have suggested that the thymus plays a critical role in the pathogenic events leading to cGVHD 3 4 5 6 7 8 9 10. The thymus, especially thymic epithelial cells (TECs), is one of the primary targets of graft‐versus‐host disease (GVHD) 3 4 5 6 7 8 9 10 11 12 13 14 15 16 17. The damage in TECs not only affects negative T‐cell selection, but it also impairs the generation of regulatory T cells (Tregs), leading to the development of cGVHD 16. Therefore, strategies to restore or regenerate TECs should lead to the prevention of cGVHD 3, 18.

It is well known that embryonic stem cells (ESCs) have the dual ability to propagate indefinitely in vitro in an undifferentiated state and to differentiate into many types of cells that derived from all three germ layers 19. ESCs have been shown to be able to generate different types of cells in vitro 19. We have reported that mouse embryonic stem cells (mESCs) can be selectively induced to generate thymic epithelial progenitors (TEPs) in vitro 20. When transplanted into syngeneic and allogeneic HSCT recipients, the mESC‐TEPs further developed into cortical TECs (cTECs) and medullary TECs (mTECs), reconstituted normal thymic architecture, and promoted thymocyte generation, leading to increased numbers of functional T cells in the periphery 20, 21. Importantly, the reconstituted immune system was tolerant to host, HSCT donor, and third‐party mESC‐TEP/TEC antigens 21, 22.

In this study, we determined the ability of mESC‐TEPs to control cGVHD. We show here that transplantation of mESC‐TEPs into the thymus of cGVHD recipients results in immune tolerance to donor and host antigens, leading to the prevention of cGVHD.

## Materials and Methods

### Mice

BALB/c, C57BL/6 (B6), and Rag1^−/−^ mice were purchased from Jackson Laboratory (Bar Harbor, ME, https://www.jax.org). Mice were used in accordance with protocols approved by the Institutional Animal Care and Use Committee of the University of Connecticut.

### Cell Culture and Differentiation

B6 mESC and GFP^+^ B6 mESC lines (Cyagen, Santa Clara, CA, http://www.cyagen.com) were maintained on irradiated murine embryonic fibroblasts in PluriQ ES‐DMEM medium (MTI GlobalStem, Gaithersburg, MD, https://www.mti-globalstem.com) with 15% fetal bovine serum, embryonic stem cell‐qualified (Thermo Fisher Scientific Life Sciences, Carlsbad, CA, https://www.thermofisher.com) and 10^3^ U/ml leukemia inhibitory factor. For the differentiation of B6 mESCs into definitive endoderm (DE), dissociated mESCs were seeded on gelatin‐coated plates and cultured in 50% Dulbecco's modified Eagle's medium (DMEM), and 50% Advanced DMEM/F‐12 medium (Thermo Fisher) supplemented with 55 μM β‐mercaptoethanol (Thermo Fisher) and 0.2% BSA (Sigma‐Aldrich, St. Louis, MO, https://www.sigmaaldrich.com). The combination of activin A (50 ng/ml; R&D Systems, Minneapolis, MN, USA, https://www.rndsystems.com), Noggin (200 ng/ml; R&D Systems), and a GSK3β inhibitor, 1‐azakenpaullone (AKP; 2.5 μM; Sigma‐Aldrich), were added to the cultures from days 3–6 as described 23. For the differentiation of mESC‐DE into TEPs, the cells were cultured in the presence of fibroblast grown factor (FGF) 7 (20 ng/ml), FGF10 (20 ng/ml), epithelial growth factor (EGF) (50 ng/ml), and bone morphogenetic protein 4 (BMP‐4) (20 ng/ml) (R&D Systems or Pepro Tech, Rocky Hill, NJ, https://www.peprotech.com), recombinant (r) FOXN1 (100 ng/ml), and rHOXA3 protein (200 ng/ml) for another 10 days 24.

### Flow Cytometry Analysis

Single‐cell suspensions of the thymus, spleen, graft, and mESC‐derived cells were stained with the fluorochrome‐conjugated antibodies as described 25, 26. For intracellular staining, the cells were first permeabilized with a BD Cytofix/Cytoperm solution for 20 minutes at 4°C. Direct or indirect staining of fluorochrome‐conjugated antibodies included CD4, CD8, CD3, CD25, Foxp3, CD45, Ly51, and EpCAM1 (BioLegend or BD Biosciences, San Diego, CA, http://www.bdbiosciences.com), keratin (k)5 (SantaCruz Biotechnology, Santa Cruz, CA, http://www.scbt.com), k8 (US Biological, Swampscott, MA, https://www.usbio.net), fluorescein isothiocyanate, phycoerythrin‐labeled anti‐rat or ‐rabbit IgG (BD Biosciences). The samples were analyzed on a FACSCalibur machine (BD Biosciences). Data analysis was performed using FlowJo software (Ashland, OR, http://www.flowjo.com).

### Real‐time Quantitative Reverse‐Transcription and Polymerase Chain Reactions

Total RNA was isolated from cells, and cDNA was synthesized as described 27. Quantitative reverse‐transcription polymerase chain reaction (qRT‐PCR) was performed with the Power SYBR green mastermix (Thermo Fisher) using the 7500 real‐time polymerase chain reaction (PCR) system (Thermo Fisher). Primers are summarized in supplemental online Table 1.

### Immunomagnetic Cell Separation

Single‐cell suspensions from differentiated mESCs were harvested after the cells were treated with 2 mg/ml collagenase IV. The cells were stained with rat anti‐mouse EpCAM1 antibody, washed, and stained with anti‐rat IgG MicroBeads (Miltenyi Biotec, Auburn, CA, http://www.miltenyibiotec.com). EpCAM1^+^ and EpCAM1^−^ cells were selected using a magnetic‐activated cell sorter immunomagnetic separation system (Miltenyi Biotec).

### Intrathymic Injection

The thymus was surgically exposed and one‐half of the indicated number of mESC‐derived cells were injected into the anterior superior portion of each lobe (10 μl per site) using a 0.1‐ml syringe (with attached 28‐gauge needle), as described 20, 21. Control mice were injected intrathymically (i.t.) with phosphate‐buffered saline (PBS) alone.

### HSCT Procedure

Bone marrow (BM) was harvested from mice by flushing the marrow from the femurs and tibias with cold RPMI 1640 supplemented with sodium bicarbonate (2 mg/ml) and 1% 4‐(2‐hydroxyethyl)‐1‐piperazineethanesulfonic acid. T‐cell‐depleted (TCD) BM cells were prepared by incubating the BM cell suspensions with anti‐Thy1.2 antibody for 30 minutes at 4°C, followed by incubation with low‐TOX‐M rabbit complement (Cedarlane Laboratories, Hornby, ON, Canada, https://www.cedarlanelabs.com), as described 25. Recipients received 900 cGy total body irradiation from a 137Cs source (Gammator‐50 γ Irradiator; Radiation Machinery Corporation, Parsippany, NJ). Two to four hours later, the mice were injected intravenously (i.v.) with TCD‐BM (5 × 10^6^) with or without splenic cells (1.25 × 10^6^) or purified splenic T cells (0.1–0.5 × 10^6^), and i.t. with mESC‐derived cells (5 × 10^4^).

### Assessment of GVHD

Clinical cutaneous cGVHD scores were assessed as described 28. In brief, the evaluation was based on the area of alopecia as follows: 0.5: skin ulceration but no hair loss; 1: skin ulcer with alopecia, 1 cm^2^ in area; 2: skin ulcer with alopecia 1–3 cm^2^; 3: skin ulcer with alopecia15% body area; and 4: skin ulcer with alopecia 30% body area.

cGVHD mice were euthanized on day 60 after HSCT, and cGVHD target organs were harvested for histopathological analysis. The organs were formalin preserved, paraffin embedded, sectioned, and hematoxylin/eosin (H&E) stained. Assessment of tissue damage was performed based on scoring systems previously described 28. In brief, gut cGVHD was scored on the basis of crypt apoptosis and lamina propria inflammation; the maximum score is 8. Skin tissue cGVHD was scored on the basis of tissue damage in the epidermis and dermis, especially expansion of dermis with collagen deposition and loss of subcutaneous fat; the maximum score is 9. Salivary gland tissue cGVHD was scored on mononuclear cell infiltration and structural disruption, with a maximum score of 8. To assess collagen in the skin, tissues were stained with Trichrome Stain Kit (Sigma‐Aldrich).

### Kidney Capsule Grafting and Bone Marrow Transplantation

Purified mESC‐derived EpCAM1^+^ or EpCAM1^−^ cells were subjected to reaggregate cultures for 24–48 hours, as described 20. Mice were transplanted under the kidney capsule with the solidified reaggregate of the mESC‐derived cells.

### Immunohistology and Confocal Microscopy

Immunohistological analysis of grafts from kidney capsule was performed according to a modified protocol 20. Briefly, the graft tissues were incubated in 4% paraformaldeyde for 4 hours followed by incubation in 30% sucrose solution overnight. The tissues were embedded in optimal cutting temperature medium, snap frozen, and subsequently cut into 5‐µm sections. The sections were stained with rabbit anti‐mouse K5 polyclonal antibody (Covance Research Products, Denver, PA, http://www.covance.com), and rat anti‐mouse K8 monoclonal antibody (Troma‐I mAb, raised by P. Brulet and R. Kemler and obtained from the Developmental Studies Hybridoma Bank, University of Iowa, IA). Serum autoantibodies were examined by staining Rag‐1^−/−^ murine skin and salivary gland tissues as described 3. In brief, cryosections of the tissues were prepared, blocked with 5% bovine serum albumin in PBS for 2 hours, incubated overnight with 5×‐diluted serum from cGVHD recipients, and then incubated with secondary antibody and DAPI. Cells of mESC‐DE in slides were stained with goat anti‐Foxa2 antibody (Santa Cruz Biotechnology) and anti‐Sox17 monoclonal antibody (R&D Systems). The secondary antibodies used were AlexaFluor‐488‐, or 594‐conjugated anti‐mouse, ‐rat, ‐rabbit, or ‐goat IgG (Thermo Fisher). The sections and cells were observed under a Nikon A1R confocal microscope (Nikon, Kanagawa, Japan, http://www.nikon.com).

### Cytokine Analysis

Blood samples were obtained from cGVHD mice on day 60 after HSCT. Cytokine content was measured using the Cytometric Bead Array (CBA)‐Mouse Th1/Th2 cytokine kit, the CBA Mouse IL‐17A Enhanced Sensitivity Flex Set, and Mouse Enhanced Sensitivity Master Buffer Kit (BD Biosciences) as described previously 22.

### Mixed Leukocyte Reactions

Splenocytes (normalized to 1 × 10^5^ T cells per well) from HSCT recipients were cultured in the presence or absence of irradiated (2,000 cGy) splenocytes (2 × 10^5^ cells per well) from different mouse strains in a 96‐well plate for 4 days. Cell proliferation was measured by BrdU Labeling and Detection Kit III (Roche Applied Science, Mannheim, Germany, https://lifescience.roche.com) according to the manufacturer's instructions. Absorbance (measured as optical density [OD]), proportional to BrdU uptake, was measured at 405 nm using an ELISA microplate reader (BioTek, Winooski, VT, http://www.biotek.com). The data are expressed as stimulation index (OD in mixed leukocyte reactions [MLRs]/OD in spontaneous proliferation).

For one‐way MLRs, splenocytes from HSCT recipients were depleted for CD25^+^ T cells before MLR. Briefly, splenocytes were incubated with anti‐CD25‐PE, and then anti‐PE‐labeled microbeads (Miltenyi Biotec). Depletion of CD25^+^ cells was achieved by using a magnetic‐activated cell sorter immunomagnetic separation system (Miltenyi Biotec) as described 29. The purity of the depletion using this procedure, assessed by flow cytometry, was >97%.

### Statistical Analysis

*p* values were based on the two‐sided Student's *t* test. A confidence level above 95% (*p* < .05) was determined to be significant.

## Results

### Inducing the Differentiation of mESCs From B6 Mice Into TEPs In Vitro

**Figure 1:**
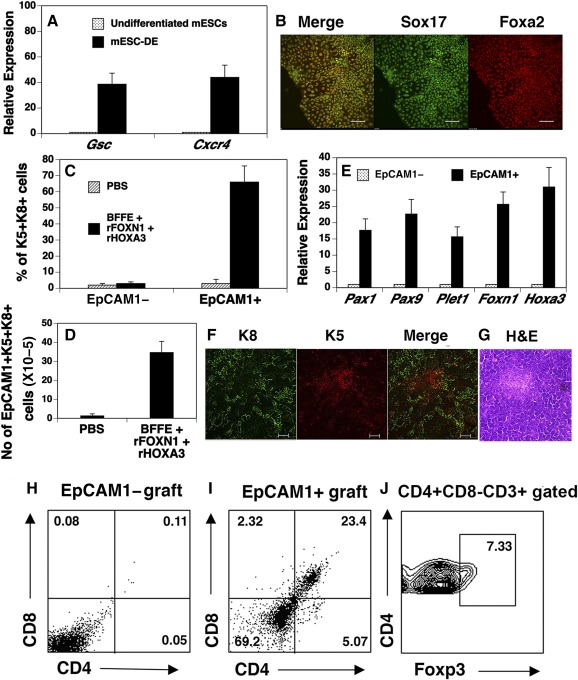
Generation of thymic epithelial progenitors from B6 mESCs in vitro. **(A, B):** Dissociated mESCs (5 × 10^5^ cells per well) were seeded on gelatin‐coated plates. Activin A (50 ng/ml), Noggin (200 ng/ml), and 1‐azakenpaullone (2.5 µM) were added to the cultures from days 3–6 **(A)**. On day 6, the expression of *Gsc* and *Cxcr4* was analyzed by quantitative reverse‐transcription polymerase chain reaction (qRT‐PCR). Data are presented as relative levels of expression in mESC‐derived cells versus undifferentiated mESCs. **(B):** Representative image of immunofluorescence shows the expression of Sox17 and Foxa2. Scale bar = 100 µm. **(C, D):** The mESC‐DE were cultured with PBS control or BFFE + rFOXN1 (100 ng/ml) + rHOXA3 (200 ng/ml). On day 16, **(C)** the percentage and **(D)** the number of cells that coexpressed K5 and K8 by mESC‐derived **(C, D)** EpCAM1^+^ and **(C)** EpCAM1^−^ cells was analyzed by flow cytometry. **(E):**The expression of *Pax1*, *Pax9*, *Plet1*, *Foxn1*, and *Hoxa3* in mESC‐EpCAM1^+^ cells that had been cultured with BFFE + rFOXN1+ rHOXA3 was analyzed by qRT‐PCR. Expression levels for each gene were normalized to the housekeeping gene *glyceraldehyde‐3‐phosphate dehydrogenase* and are presented as relative expression compared with mESC‐EpCAM1^−^ cells. **(F–J):** EpCAM1^+^ or EpCAM1^−^ cells were purified from day 16 mESC cultures that contained BFFE, rFOXN1, and rHOXA3. The cells were reaggregated in vitro and then transplanted under the kidney capsule of B6 mice. Two months later, **(F)** the grafts were stained with fluorochrome‐labeled anti‐K8 and anti‐K5 antibodies. A representative EpCAM1^+^ graft is shown. Scale bar = 50 µm. **(G):** A representative EpCAM1^+^ graft with H&E staining (magnification, ×200). **(H–J):** T‐cell development in the grafts was analyzed. Representative flow cytometric profiles of CD4 and CD8 double positive and single positive in **(H)** EpCAM1^−^ and **(I)** EpCAM1^+^ cell grafts, as well as **(J)** CD4^+^CD8^−^CD3^+^Foxp3^+^ regulatory T cells in EpCAM1^+^ cell graft. Data show one of three independent experiments with similar results. Abbreviations: DE, definitive endoderm; H&E, hematoxylin/eosin; mESC, mouse embryonic stem cell; PBS, phosphate‐buffered saline.

We have previously described protocols to induce the differentiation of TEPs from a TC‐1 mESC line that was derived from 129SVEVTac mice 20. However, the protocols could not efficiently induce the differentiation of mESCs from B6 mice into TEPs in vitro (data not shown). Therefore, we modified our differentiation protocols. Because TECs originate from the endoderm 30, we first induced the differentiation of B6 mESCs into DE by the combination of activin A, Noggin, and a GSK3β inhibitor AKP as described 23. Six days later, the cells were analyzed for the expression of DE markers *Gsc* and *Cxcr4* 23 by qRT‐PCR. As shown in Figure 1A, the expression of these markers was significantly increased as compared with undifferentiated mESCs. Immunofluorescence analysis also showed that most of the mESC‐derived cells coexpressed the Sox17 and Foxa2 proteins (Fig. 1B). These results indicate that the DE had been generated from the mESCs.

We then directed the differentiation of the mESC‐DE into TEPs. We have previously reported that the combination of BMP‐4, FGF7, FGF10, and EGF (BFFE) induces the differentiation of TC‐1 mESCs into TEPs 20, 21. Studies have shown that Hoxa3 and Foxn1 are critical regulators for thymus organogenesis and thymic epithelium development 31, 32. We have recently shown that rHOXA3 protein and rFOXN1 protein that was fused to the HIV transactivator of transcription protein transduction domain significantly enhance the differentiation of human ESC‐derived DE into TEPs 24. Therefore, in addition to the four growth factors, rHOXA3 and rFOXN1 were also added to mESC differentiation cultures.

We analyzed for the expression of EpCAM1 because it has been shown to be expressed by TEPs and because we have previously used it as a marker for mESC‐TEPs 20, 33. However, unlike TC‐1 mTECs, a significant percentage (54%–78%) of undifferentiated B6 mESCs expressed EpCAM1. After differentiation, the percentage of EpCAM1^+^ cells did not significantly differ from the undifferentiated mESCs. We then examined the expression of K5 and K8 from mESC‐derived cells, because it has been reported that K5 and K8 double positive (K5^+^K8^+^) cells contain or represent TEPs 34, 35. As shown in Figures 1C and 1D, the percentage and number of K5^+^K8^+^ cells in day 16 mESC‐EpCAM1^+^ cells was tremendously increased in the cultures containing BFFE, rHOXA3 and rFOXN1, as compared with cultures without all of these factors. In both of the culture conditions, few mESC‐EpCAM1^−^ cells were K5^+^K8^+^ cells (Fig. 1C). We then analyzed for the expression of TEP‐related genes *Pax1*, *Pax9*, *Plet1*, *Foxn1*, and *Hoxa3* by qRT‐PCR. The expression of these genes in mESC‐EpCAM1^+^ cells that had been cultured with BFFE, rHOXA3, and rFOXN1 was markedly higher than that in mESC‐EpCAM1^−^ cells (Fig. 1E).

To determine whether mESC‐derived cells can develop into TECs in vivo, we purified mESC‐derived EpCAM1^+^ and EpCAM1^−^ cells from the cultures of mESCs having had the addition of BFFE, rHOXA3, and rFOXN1. The cells were reaggregated in vitro and then transplanted under the kidney capsule of syngeneic B6 mice. Two months later, the grafts were harvested and analyzed for structure by immunofluorescence. Discrete K8^+^K5^−^ cortical (green color) and K8^−^K5^+^ medullary (red color) areas were present in the EpCAM1^+^ cell grafts, but not in the EpCAM1^−^ cell grafts (Fig. 1F, and data not shown). H&E staining also shows a demarcation between cortical and medullary regions in the EpCAM1^+^ cell grafts (Fig. 1G). Furthermore, CD4 and CD8 double positive (DP) and single positive (SP) T cells were generated in the EpCAM1^+^ grafts, but not in the mESC‐EpCAM1^−^ grafts (Figs. 1H, 1I). In addition, CD4^+^Foxp3^+^ Tregs were observed in the EpCAM1^+^ cell‐transplanted mice (Fig. 1J).

Taken together, these results suggest that B6 mESCs can also be selectively induced to generate TEPs in vitro. The mESC‐TEPs in vivo further developed into TECs that attract mouse T‐cell precursors to the grafts and support their development into T cells, including Tregs.

### Transplantation of Donor‐Origin mESC‐TEPs Into the Thymus of cGVHD Recipients Prevents the Development of cGVHD

**Figure 2:**
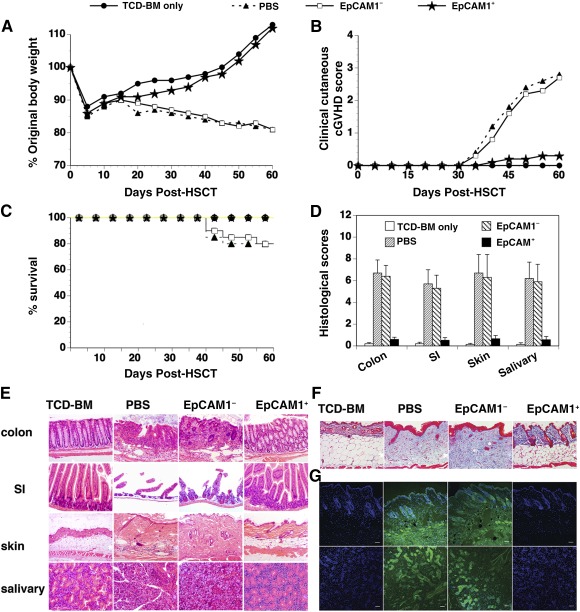
Transplantation of mESC‐thymic epithelial progenitors prevents the development of cGVHD. Lethally irradiated BALB/c recipients were injected intravenously with TCD‐BM cells and spleen cells from B6 mice and intrathymically with B6 mESC‐derived EpCAM1^+^, EpCAM1^−^ cells, or PBS on day 0. Recipients given TCD‐BM alone were used as a control. Recipients were monitored for **(A)** body weight change, **(B)** clinical cutaneous cGVHD score, and **(C)** survival. Pooled data from three separate experiments are shown, with four to five mice per group in each experiment. **(D–G):** On day 60 after HSCT, **(D, E)** recipients were analyzed for histologic damage of colon, SI, skin, and salivary gland by H&E staining. **(D)**: Mean ± SD of histopathology scores and **(E)** representative H&E staining photomicrographs. **(F):** Skin tissues were stained with trichrome for collagen (blue color) (magnification, ×200). **(G):** Recipient serum samples were tested for the presence of autoantibodies by staining donor‐type Rag‐1^−/−^ skin (top) and salivary gland (bottom) tissues. 4′,6‐diamidino‐2‐phenylindole staining is shown in blue, and autoantibody staining is shown in green. Representative photomicrographs are shown. Scale bar = 50 µm. Abbreviations: BM, bone marrow; cGVHD, chronic graft‐versus‐host disease; H&E, hematoxylin/eosin; HSCT, hematopoietic stem cell transplantation; mESC, mouse embryonic stem cell; PBS, phosphate‐buffered saline; SI, small intestine; TCD, T‐cell‐depleted.

Because TEC damage is sufficient to initiate the generation of autoreactive T cells and the development of cGVHD, we investigated whether transplantation of mESC‐TEPs prevents the development of cGVHD. We used a cGVHD model that was recently described by Zeng's group 3. In this model, cGVHD is induced by transferring a low dose of splenic cells or CD8^+^ T cells from B6 mice into BALB/c recipients. Lethally irradiated BALB/c recipients were injected i.v. with TCD‐BM and a low dose of spleen cells from B6 mice, and i.t. with B6 mESC‐EpCAM1^+^ TEPs, mESC‐EpCAM1^−^ cells, or PBS. Mice that were injected with TCD‐BM only were used as cGVHD negative controls. As shown in Figures 2A, 2B, and 2C, PBS‐ or mESC‐EpCAM1^−^ cell‐treated mice developed gradual body weight loss and hair loss although 80% of these mice still survived for more than 60 days. In contrast, transplantation of B6 mESC‐EpCAM1^+^ TEPs prevented this body weight loss and hair loss with all mice surviving for more than 60 days.

Histopathologically, PBS‐ or mESC‐EpCAM1^−^ cell‐treated mice manifested typical cGVHD characteristics including expansion of dermis with collagen deposition and loss of subcutaneous fat in the skin, infiltration and loss of crypts in the guts, and infiltration and destruction of secretory follicles in the salivary gland (Figs. 2D–F). In contrast, there were fewer inflammatory cell infiltrates and less tissue damages in mESC‐TEP‐treated cGVHD recipients. Consequently, the histological scores in mESC‐TEP‐treated recipients were significantly reduced, as compared with PBS‐treated recipients (Fig. 2D).

Furthermore, immunofluorescent staining revealed that sera from PBS‐ or mESC‐EpCAM1^−^ cell‐treated cGVHD recipients on day 60 after HSCT had strong autoantibody staining of donor‐type and recipient‐type Rag1^−/−^ skin and salivary gland tissues, whereas sera from mESC‐EpCAM1^+^ TEP‐treated recipients did not (Fig. 2G, and data not shown).

Several studies have shown that cGVHD is associated with increased inflammatory cytokines, such as interleukin‐6 (IL‐6), tumor necrosis factor‐α (TNF‐α), and interleukin‐17A (IL‐17A) 36, 37. We then examined Th1/Th2/Th17 cytokines in the sera. We found that IL‐6, TNF‐α, and IL‐17A were elevated in control‐treated cGVHD recipients, but they were significantly reduced in mESC‐EpCAM1^+^ TEP‐treated recipients ( supplemental online Fig. 1, and data not shown).

Similar results were obtained in mESC‐TEP‐treated cGVHD recipients that were induced by purified CD8^+^ T cells 3 from B6 mice (data not shown). Taken together, our results suggest that transplantation of mESC‐TEPs into allogeneic HSCT recipients prevents the development of cGVHD.

### mESC‐TEP‐Treated cGVHD Recipients Have Increased Numbers of TECs and Thymocytes

**Figure 3:**
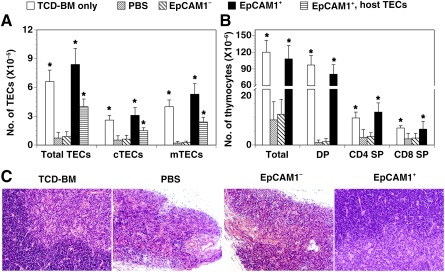
mESC‐thymic epithelial progenitors‐treated chronic graft‐versus‐host disease recipients have more TECs and thymocytes. Lethally irradiated BALB/c recipients were injected intravenously with TCD‐BM cells and spleen cells from B6 mice, and intrathymically with GFP^+^ B6 mESC‐derived EpCAM1^+^ cells, EpCAM1^−^ cells, or PBS on day 0. Recipients given TCD‐BM alone were used as a control. On day 60 after transplant, the number of **(A)** total and host‐origin (GFP^−^) TECs (CD45^−^EpCAM1^+^), cTECs (CD45^−^EpCAM1^+^Ly51^+^), mTECs (CD45^−^EpCAM1^+^Ly51^−^), and **(B)** total, CD4 and CD8 DP, CD4 SP and CD8 SP thymocytes was analyzed by flow cytometry. The data are expressed as mean ± SD from one of three independent experiments with similar results (four to five mice per group in each experiment). **(C):** Representative hematoxylin/eosin staining photomicrographs for the thymus from indicated groups (magnification, ×200). ∗, *p* < .05, compared with PBS‐treated mice. Abbreviations: BM, bone marrow; cTECs, cortical thymic epithelial cells; DP, double positive; mESC, mouse embryonic stem cell; mTECs, medullary thymic epithelial cells; PBS, phosphate‐buffered saline; SP, single positive; TCD, T‐cell‐depleted; TECs, thymic epithelial cells.

Our previous studies have shown that transplantation of mESC‐TEPs into non‐GVHD recipients results in more TECs and thymocytes 20, 21. We determined whether this would also occur in GVHD recipients. As shown in Figure 3A, PBS‐ or mESC‐EpCAM1^−^ cell‐treated cGVHD mice had reduced numbers of total TECs (CD45^−^EpCAM1^+^), and their subsets cTECs (CD45^−^EpCAM1^+^Ly51^+^) and mTECs (CD45^−^EpCAM1^+^Ly51^−^). In contrast, transplantation of mESC‐EpCAM1^+^ TEPs resulted in more total TECs, cTECs, and mTECs, consistent with our previous data in non‐GVHD models 20, 21. Interestingly, by using GFP^+^ B6 mESCs to separate mESC‐TECs and host TECs, we found that the number of host TECs (GFP^−^) and their subsets cTECs and mTECs was also significantly increased in mESC‐TEP‐transplanted cGVHD recipients, as compared with control‐treated recipients (Fig. 3A). The host TECs and mESC‐derived TECs (total, cTECs, and mTECs) were in approximately a 50/50 ratio.

We then analyzed thymocytes. The number of total and CD4 and CD8 DP thymocytes in PBS‐ or mESC‐EpCAM1^−^ cell‐treated cGVHD mice was tremendously reduced, as compared with non‐GVHD mice (recipients received TCD‐BM only). In contrast, transplantation of mESC‐EpCAM1^+^ TEPs significantly increased the number of total, DP, and CD4 SP and CD8 SP thymocytes to a level comparable with that in the non‐GVHD mice (Fig. 3B).

We also examined the thymic architecture. Similar to TCD‐BMT only group, the thymus from mESC‐EpCAM1^+^ TEP recipients shows a sharp demarcation between cortical regions in H&E staining. In contrast, atrophic thymus from PBS‐ or mESC‐EpCAM1^−^ cell‐treated cGVHD mice displayed disorganized boundaries between cortex and medulla (Fig. 3C).

### mESC‐TEP‐Treated cGVHD Recipients Have Increased Number of Tregs

**Figure 4:**
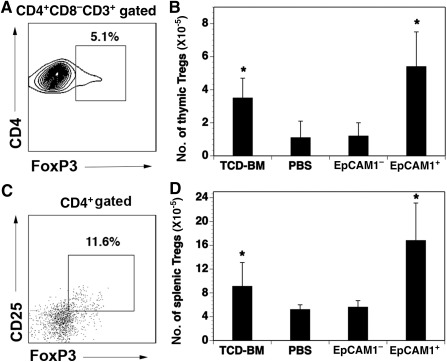
mESC‐TEP‐treated chronic graft‐versus‐host disease recipients have more Tregs. Lethally irradiated BALB/c recipients were injected intravenously with TCD‐BM and spleen cells from B6 mice and intrathymically with mESC‐derived EpCAM1^+^ cells, EpCAM1^−^ cells, or PBS, as in Figure 2. On day 60 after transplant, Tregs in the thymus and spleen were analyzed by flow cytometry. **(A):** A representative flow cytometric profile showing thymic Tregs in mESC‐EpCAM1^+^ TEP recipients. **(B):** The number of thymic CD4^+^CD8^−^CD3^+^FoxP3^+^ Tregs from each group. **(C):** A representative flow cytometric profile showing the Tregs in the spleen of mESC‐EpCAM1^+^ TEP recipients. **(D):** The number of splenic CD4^+^CD25^+^FoxP3^+^ Tregs from each group. **(C, D):** The data are expressed as mean ± SD from one of three independent experiments with similar results (four to five mice per group in each experiment). ∗, *p* < .05, compared with PBS‐treated mice. Abbreviations: BM, bone marrow; mESC, mouse embryonic stem cell; PBS, phosphate‐buffered saline; TCD, T‐cell‐depleted; TEP, thymic epithelial progenitor; Tregs, regulatory T cells.

Accumulating data have shown that Tregs also play an important role in the prevention of GVHD 38 39 40 41 42 43 44 45. Because TECs can support Treg development, we analyzed Tregs in the cGVHD recipients. As shown in Figures 4A–4D, the numbers of Tregs in the thymus and spleen of mESC‐TEP‐treated cGVHD recipients were significantly higher than that in PBS‐ or mESC‐EpCAM1^−^ cell‐treated cGVHD mice. It is likely that both host TECs and mESC‐TECs support the development of the thymic Tregs, leading to the increased number of Tregs in the periphery.

### Transplantation of mESC‐TEPs Into the Thymus of cGVHD Recipients Results in Immune Tolerance to Both the Donor and Host Antigens

**Figure 5:**
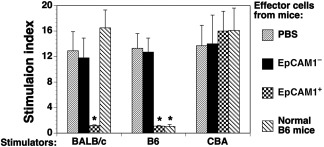
T cells from mouse embryonic stem cell‐ thymic epithelial progenitor‐treated chronic graft‐versus‐host disease recipients fail to proliferate in response to donor or host antigens but were able to mount an immune response to third‐party antigens. Lethally irradiated BALB/c recipients were injected intravenously with thymic epithelial progenitors‐bone marrow and spleen cells from B6 mice and intrathymically with B6 mESC‐derived EpCAM1^+^ cells, EpCAM1^−^ cells, or PBS, as in Figure 2. On day 60 after transplant, splenocytes from the recipients were harvested and used as effector cells for mixed leukocyte reactions. The effector cells were cultured with irradiated splenocytes (as stimulators) from normal non‐hematopoietic stem cell transplantation BALB/c, B6, and CBA mice, respectively. Cell proliferation was determined. Data are shown as stimulation index. The data are expressed as mean ± SD from one of three independent experiments with similar results (four to five mice per group in each experiment). ∗, *p* < .05, compared with PBS‐treated mice. Abbreviations: CBA, cytometric bead array; PBS, phosphate‐buffered saline.

To determine the mechanisms by which transplantation of mESC‐TEPs prevents cGVHD development, we investigated whether immune tolerance to the donor and host allo‐antigens had been established in mESC‐TEP‐transplanted cGVHD recipients. MLR showed that splenocytes from control‐ (PBS or mESC‐EpCAM1^−^ cell)‐treated mice proliferated robustly in response to the donor and host antigens, whereas those from mESC‐EpCAM1^+^ TEP‐treated mice did not (Fig. 5). In contrast, splenocytes from all animal groups were able to mount an immune response to third‐party antigens (from CBA mice). These results suggest that T cells in mESC‐TEP‐treated cGVHD recipients are immune tolerant to both donor and host allo‐antigens.

Because we have shown that the number of Tregs was increased in mESC‐TEP‐treated cGVHD recipients, we wanted to determine whether the Tregs play a role in the immune tolerance. We used one‐way MLR in which splenocytes from the cGVHD recipients were depleted for CD25^+^ Tregs before MLR. The deletion of CD25^+^ Tregs resulted in splenocytes from mESC‐TEP‐treated cGVHD recipients proliferating in response to the donor and host allo‐antigens ( supplemental online Fig. 2). The results suggest that Tregs in mESC‐TEP‐treated cGVHD recipients play a role in the immune tolerance. However, the response levels of CD25^+^ Treg‐depleted splenocytes from mESC‐TEP‐treated cGVHD recipients were still lower than those from PBS or mESC‐EpCAM1^−^ cell‐treated cGVHD recipients ( supplemental online Fig. 2).

## Discussion

Despite the advance in transplantation practices, cGVHD remain to be a major limiting factor for a successful allogeneic HSCT 2, 3, 46, 47. This is mainly due to our failure to reduce the incidence and severity of cGVHD 2. In addition to GVHD, patients following HSCT often suffer a prolonged period of T‐cell reconstitution from donor HSCs, resulting in a profound T‐cell immunodeficiency, which is associated with an increased risk of infections, and occurrence or relapse of cancers. GVHD, pre‐HSCT conditioning, and the age‐dependent thymus contribute to the prolonged T‐cell regeneration. It has also been reported that T‐cell immunodeficiency itself increases the susceptibility to GVHD 48. Therefore, developing a therapeutic strategy to prevent GVHD without compromising the immune system would be ideal for allogeneic HSCT recipients.

TECs are the major component of the thymic microenvironment for T‐cell development 30, 49, 50. TECs play a critical role in immune tolerance induction by mediating negative selection and supporting Treg generation. Unfortunately, TECs are one of the primary targets of GVHD 3 4 5 6 7 8 9 10 11 12 13 14 15 16 17. Because damage in TECs is sufficient to induce cGVHD, we hypothesized that restoration or regeneration of TECs could prevent the development of cGVHD. Indeed, we show here that transplantation of donor‐origin mESC‐TEPs into the thymus of cGVHD recipients induces immune tolerance to both donor and host allo‐antigens, leading to the prevention of cGVHD. Furthermore, T‐cell regeneration in the thymus was significantly enhanced in mESC‐TEP‐treated recipients, which can overcome T‐cell immunodeficiency following allogeneic HSCT.

Interestingly, in addition to the generation of mESC‐derived TECs, the number of host TECs was also significantly increased. This is important for inducing immune tolerance to host antigens because, in addition to being damaged by GVHD, TECs undergo both a qualitative and quantitative loss with age 49, 50, which contributes to the increased incidence of autoimmunity diseases among older adults 51. The mechanisms by which transplantation of mESC‐TEPs results in more host TECs remain to be investigated. It is possible that mESC‐TEPs and/or TECs directly contact with host TECs or produce growth factors, such as FGF7 52, 53, to stimulate host TECs.

Our MLR assay shows that T cells from mESC‐TEP‐transplanted mice failed to proliferate in response to stimulation with donor and host allo‐antigens but were able to mount an immune response to third‐party antigens. Studies have shown that Tregs play an important role in maintaining peripheral tolerance and prevention of GVHD 38 39 40 41 42 43 44 45 and that TECs support the development of Tregs 54, 55. We have shown that the number of Tregs is increased in mESC‐TEP‐treated cGVHD recipients. Our one‐way MLR suggests that the Tregs play a role in the immune tolerance to donor and host allo‐antigens. Because the response levels of Treg‐depleted splenocytes from mESC‐TEP‐treated cGVHD recipients were still lower than those from control‐treated cGVHD recipients, it is likely that both the mESC‐TECs and the host TECs also contribute to negative selection, leading to, at least in part, the deletion or inactivation of reactive T cells for donor and host allo‐antigens.

Studies have shown that B cells also play an important role in the development of cGVHD 46, 56 and that Tregs can inhibit autoantibody producing‐plasma cells in cGVHD recipients 45. Our data show that control‐treated cGVHD recipients contained donor and recipient specific autoantibody, whereas mESC‐TEP‐treated mice did not. It will be of interest to investigate whether this is due to the inhibition of autoantibody producing‐plasma cells by Tregs in mESC‐TEP‐treated mice.

## Conclusion

We have demonstrated that transplantation of mESC‐TEPs can prevent the development cGVHD. Therefore, generation of TEPs from human ESCs have the potential to provide a novel approach to prevent the development of cGVHD in patients following allogeneic HSCT.

## Author Contributions

R.H.: collection and/or assembly of data, data analysis and interpretation, manuscript writing; Y.L., M.S., and Y.S.: collection and/or assembly of data, data analysis and interpretation; D.R.: administrative support, collection and/or assembly of data; L.L.: conception and design, financial support, collection and/or assembly of data, data analysis and interpretation, manuscript writing, final approval of manuscript.

## Disclosure of Potential Conflicts of Interest

The authors indicated no potential conflicts of interest.

## Supporting information

Supporting Information
