# Supplementary material for: Transplantation of Donor‐Origin Mouse Embryonic Stem Cell‐Derived Thymic Epithelial Progenitors Prevents the Development of Chronic Graft‐versus‐Host Disease in Mice
Source: Stem Cells Transl Med. 2016 Aug 2;6(1):121–30. doi: 10.5966/sctm.2016-0012 (PMC5442732; doi:10.5966/sctm.2016-0012)
Supplement: Supplementary file 1 — Supporting Information [file SCT3-6-121-s001.pdf]

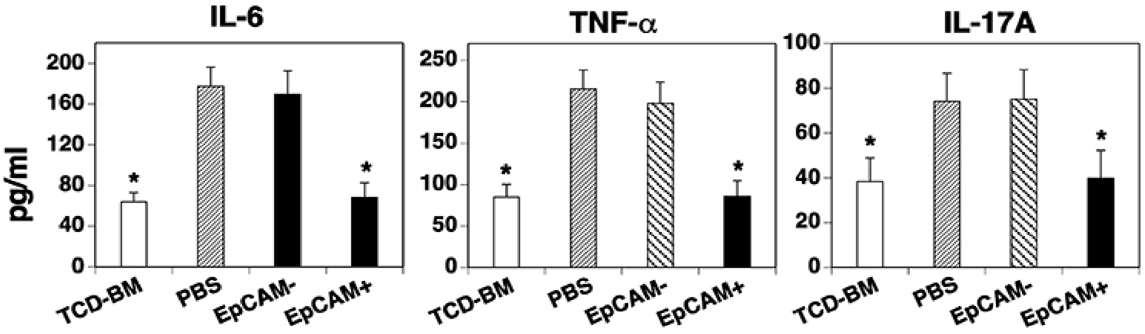

Supplemental Figure 1. mESC-TEP-treated cGVHD recipients have a reduced production of proinflammatory cytokines. Lethally irradiated BALB/c recipients were injected i.v. with TCD-BM cells and spleen cells from B6 mice and i.t. with B6 mESC-derived EpCAM1$^+$, EpCAM1$^-$ cells, or PBS on day 0. Recipients given TCD-BM alone were used as a control. On day 60 after HSCT, the sera were harvested and analyzed for cytokine content. The data are expressed as mean ± SD from one of two independent experiments with similar results (4-5 mice per group in each experiment). * P<0.05, compared with PBS-treated cGVHD recipients.

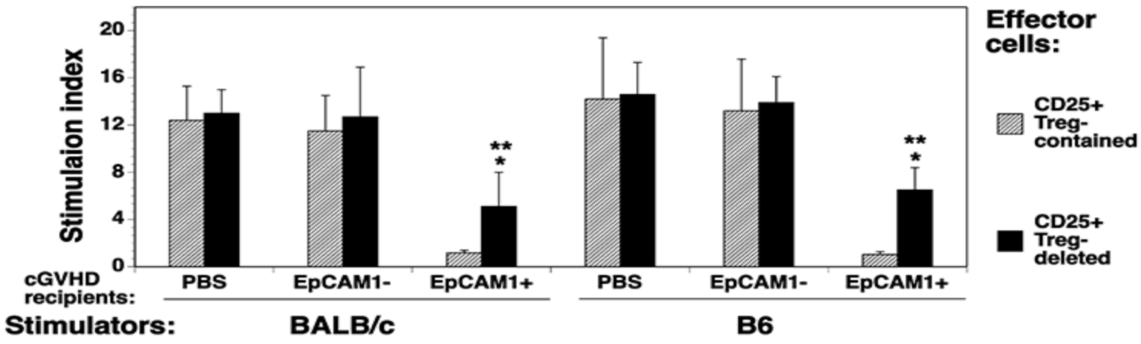

Supplemental Figure 2. Tregs in mESC-TEP-treated cGVHD recipients play a role in the immune tolerance to donor and host antigens. Lethally irradiated BALB/c recipients were injected i.v. with TCD-BM and spleen cells from B6 mice and i.t. with B6 mESC-derived EpCAM1$^+$, EpCAM1$^-$ cells, or PBS as in Figure 5. On day 60 post-transplant, splenocytes were harvested from the recipients. Part of the splenocytes were depleted for CD25$^+$ Tregs. Splenocytes with or without CD25$^+$ Tregs were used as effector cells for MLR. The effector cells were cultured with irradiated splenocytes (as stimulators) from normal non-HSCT BALB/c and B6 mice, respectively. Cell proliferation was determined. Data are shown as stimulation index. The data are expressed as mean $\pm$ SD from one of two independent experiments with similar results (4-5 mice per group in each experiment). * $P<0.05$, compared with CD25$^+$ Treg-contained splenocytes from EpCAM1$^+$ cell-treated cGVHD recipients; **$P<0.05$, compared with CD25$^+$ Treg-deleted splenocytes from PBS-treated cGVHD recipients.

Supplemental Table 1. Sequences of primers used for qRT-PCR analysis

Gene	Forward Primer	Reverse Primer
gapdh	CTTCACCACCATGGAGAAGGC	GGCATGGACTGTGGTCATGAG
Gsc	ACCATCTTCACCGATGAGCAGC	CTTGGCTCGGCGGTTCTTAAAC
Cxcr4	GTAACCACCACGGCTGTAGA	AGTAGATGGTGGGCAGGAAG
Pax1	GATGGAAGACTGGGCGGGTGTGAA	TTCTCGGTGTTTGAAGGTCATTGCCG
Pax9	GGCCAGGCACCGAATG	GCCATGCTGGATGCTGAGA
Plet1	ACACCTCCGACATCTTGGAAA	GGGACCGTCACTGTATAGGTTACAT
Foxn1	CTCGTCGTTTGTGCCTGAC	TGCCTCTTGTAGGGGTGGAAA
Hoxa3	AAGTGCCAACAGCAACCCTAC	AGGGAAAGATTTGTTTGCCCA
